# Food insecurity and water insecurity measurement in Brazil: Sustainable Development Goals monitoring through experiential scales

**DOI:** 10.1590/0102-311XEN153524

**Published:** 2025-02-28

**Authors:** Rosana Salles-Costa, Camilla Christine de Souza Cherol, Poliana de Araújo Palmeira, Ana Maria Segall-Corrêa, Sera Lewise Young, Rafael Pérez-Escamilla, Juliana de Bem Lignani, Rodrigo Pinheiro de Toledo Vianna, Mayline Menezes da Mata, Maria Angélica Tavares de Medeiros, Elaine Martins Pasquim, Juliana Chaves Barbosa, Thainá Ferreira de Lima, Olga Patrícia García-Obregón, Pablo Gaitán-Rossi, Juliana Schober Gonçalves Lima, Mauro Eduardo Del Grossi, Silvia Maria Voci, Sandra Maria Chaves dos Santos, Hugo Ramiro Melgar-Quiñonez

**Affiliations:** 1 Universidade Federal do Rio de Janeiro, Rio de Janeiro, Brasil.; 2 Universidade Federal de Campina Grande, Campina Grande, Brasil.; 3 Fiocruz Brasília, Brasília, Brasil.; 4 Northwestern University, Evanston, U.S.A.; 5 Yale University, New Haven, U.S.A.; 6 Universidade do Estado do Rio de Janeiro, Rio de Janeiro, Brasil.; 7 Universidade Federal da Paraíba, João Pessoa, Brasil.; 8 Universidade Federal de São Paulo, Santos, Brasil.; 9 Universidade Federal do Amazonas, Manaus, Brasil.; 10 Ministério da Ciência, Tecnologia e Inovação, Brasília, Brasil.; 11 Universidad Autónoma de Querétaro, Juriquilla, México.; 12 Universidad Iberoamericana Ciudad de México, Ciudad de México, México.; 13 Universidade Federal de Sergipe, São Cristóvão, Brasil.; 14 Universidade de Brasília, Brasília, Brasil.; 15 Universidade Federal da Bahia, Salvador, Brasil.; 16 McGill University, Montreal, Canada.

The 2030 Agenda for Sustainable Development ^1^ underscores the urgency of addressing critical global challenges. Among these, the 2nd Sustainable Development Goal (SDG) aims to end hunger, achieve food security and improve nutrition ^1^. Thus, experience-based measurement scales are useful instruments to monitor SDGs and support the development of policies and programs by governmental institutions.

Brazil has a successful history of validating and using a food insecurity scale to monitor hunger via national surveys ^2^, especially among *quilombolas* ^3^ and Brazilian Indigenous people ^4^. The *Brazilian Food Insecurity Scale* (EBIA, acronym in Portuguese) has been used in the last two decades ^5^, detecting a reduction in moderate and severe food insecurity between 2004 and 2014, as well as its resurgence at alarming rates during the COVID-19 pandemic ^5^. This indicates that sharp food insecurity fluctuations in Brazil corresponds with changes in political administrations with very different and even opposing social protection policies and agendas ^6^. EBIA findings have led to an increase in social awareness and mobilization to hold governments accountable for food insecurity increase and influenced food and nutrition governance at various government levels ^6^.

The Brazilian Institute of Geography and Statistics (IBGE, acronym in Portuguese) released the recent national food insecurity estimates, indicating a significant reduction after the pandemic ^7^ and changes in the political administration since 2023. However, about 8.6 million Brazilians still experience severe food insecurity ^7^, seriously compromising the achievement of SDG2, which calls for eradicating hunger ^1^.

To fulfill people’s basic rights to health and address its social determinants, including the strongly interrelated water security and food security ^8^, it is crucial to continue building from EBIA experience to provide governments, research institutes, and researchers with a valid and reliable scale to assess water insecurity in Brazil. The diversity of biomes, different sociocultural contexts and strong social inequities prevalent in our society must be considered. A more comprehensive water insecurity assessment and monitoring will help understand its relationship with food and nutrition governance, as well as how it relates to health, improving governance of both water and food systems.

Addressing the challenges of unequal water supply and increasing water scarcity are a urgent public health issue. According to the United Nations (UN) ^9^, low water availability and economic poverty negatively impact water access globally and are closely related to the SDG6, which aims to ensure availability, access and sustainable management of water and sanitation for all.

Given the worsening global water crisis due to climate change, the World Meteorological Organization (WMO) has been drawing the attention of the international community ^10^ to the global increase in extreme weather events. In Latin America/Caribbean, high temperatures, heat waves, extreme droughts, heavy rains, and floods have become more frequent and more severe ^10^. This affects water resources and consequently compromise their availability, access, and quality of water for consumption and food production, especially among vulnerable groups ^11^. Thus, climate events increase the risk of water insecurity, reinforcing inequitable access to safe water, with serious consequences for populational health, impacting food production and resulting in food insecurity ^8^. Therefore, extreme climate events associated with climate change are a threat to both food security and water security.

The Brazilian public health framework recognizes the intersectoral and interrelated nature of food insecurity and water insecurity as social determinants of health ^12^. This refers to how social conditions surrounding food insecurity and water insecurity represent social inequalities of our population, especially those in extreme poverty. It emphasizes the coordination of water and food systems’ actions related to urban and regional development, alongside sectors or policies such as housing, poverty eradication, environmental protection, health promotion, and other socially relevant issues ^13^. Given the importance of assessing water availability, the Brazilian National Water Security Plan (PNSH, acronym in Portuguese) assesses four water availability dimensions (human, economic, ecosystems, and resilience) with a Water Security Index (WSI) ^14^. This index is used to assess the country’s water supply, water security for the agricultural and industrial sectors, vulnerability of water sources for human consumption and multiple uses, in addition to the potential of natural and man created water stocks. According to WSI data ^14^, in 2017, about 61 million Brazilians had poor guarantee of water supply in cities, which is one of the water insecurity dimensions. Furthermore, the projected WSI scenario for 2035 is expected to worsen, affecting around 74 million people.

Meanwhile, the WSI does not assess water insecurity in households and how it impacts the everyday lives of its residents. Regarding traditional water indicators, we could articulate water security in four domains: water availability, accessibility, use, and reliability over time ^14^. Therefore, the use of water insecurity experiential scales could complement current indicators by measuring people’s challenges concerning water access, use and reliability, and would be very useful for the SDG debate in the 2030 Agenda.

To measure water insecurity in population studies, the quantitative experiential *Household Water Insecurity Experiences* (HWISE) scale ^8^ was proposed in 2019 by a large team of international researchers ^15^. HWISE focuses on multiple dimensions by measuring water insecurity based on residents’ experiences regarding adequacy, reliability, accessibility and safety of water in their household ^15^. Those with higher water insecurity report greater and more frequent negative experiences in the evaluated dimensions ^16^. HWISE has since been translated into dozens of languages, including Portuguese (Box 1).

**Box 1:** Items from the current English version of the *Household Water Insecurity Experiences* (HWISE) scale and the current Portuguese version of the *Brazilian Household Water Insecurity Scale*.

ENGLISH VERSION		PORTUGUESE VERSION	
LABEL	ITEMS	*CONCEITO*	*PERGUNTAS*
Worry	In the last 4 weeks, how frequently did you or anyone in your household worry you would not have enough water for all of your household needs?	*Preocupação*	*Nas últimas 4 semanas, você ou alguém de sua casa esteve incomodado, preocupado ou com medo de que você não teria água suficiente para todas as suas necessidades domésticas?*
Interrupt	In the last 4 weeks, how frequently has your main water source been interrupted or limited (e.g., water pressure, less water than expected, river dried up)?	*Interrupção*	*Nas últimas 4 semanas, com que frequência o abastecimento de água da sua principal fonte de água foi interrompido (pressão da água, menos água do que o esperado, rio secou)?*
Clothes	In the last 4 weeks, how frequently have problems with water meant that clothes could not be washed?	*Roupas*	*Nas últimas 4 semanas, com que frequência os problemas com água impediram a lavagem das roupas?*
Plans	In the last 4 weeks, how frequently have you or anyone in your household had to change schedules or plans due to problems with your water situation? (Activities that may have been interrupted include caring for others, doing household chores, agricultural work, income-generating activities, etc.)	*Planos*	*Nas últimas 4 semanas, com que frequência você ou alguém de sua família mudou a sua rotina por que teve que resolver um problema por conta da água? (As atividades que podem ter sido interrompidas incluem cuidar de outras pessoas, realizar tarefas domésticas, trabalhos agrícolas, atividades geradoras de renda, dormir etc.)*
Food	In the last 4 weeks, how frequently have you or anyone in your household had to change what was being eaten because there were problems with water (e.g., for washing foods, cooking, etc.)?	*Alimentação*	*Nas últimas 4 semanas, com que frequência você ou alguém em sua casa teve que mudar o que estava sendo comido devido a problemas com água (por exemplo, para lavar alimentos, cozinhar etc.)?*
Hands	In the last 4 weeks, how frequently have you or anyone in your household had to go without washing hands after dirty activities (e.g., defecating or changing diapers, cleaning animal dung) because of problems with water?	*Mãos*	*Nas últimas 4 semanas, com que frequência você ou alguém de sua família passou sem lavar as mãos após atividades sujas (por exemplo, defecar ou trocar fraldas, limpar esterco de animais) devido a problemas com a água?*
Body	In the last 4 weeks, how frequently have you or anyone in your household had to go without washing their body because of problems with water (e.g., not enough water, dirty, unsafe)?	*Corpo*	*Nas últimas 4 semanas, com que frequência você ou alguém em sua casa teve que ficar sem lavar o corpo por causa de problemas com água (por exemplo, água insuficiente, suja, insegura)?*
Drink	In the last 4 weeks, how frequently has there not been as much water to drink as you would like for you or anyone in your household?	*Água de beber*	*Nas últimas 4 semanas, com que frequência não houve tanta água para beber como você gostaria para você ou alguém em sua casa?*
Angry	In the last 4 weeks, how frequently did you or anyone in your household feel angry about your water situation?	*Raiva*	*Nas últimas 4 semanas, com que frequência você ou alguém de sua família sentiu raiva da situação da água?*
Sleep	In the last 4 weeks, how frequently have you or anyone in your household gone to sleep thirsty because there wasn’t any water to drink?	*Dormir com sede*	*Nas últimas 4 semanas, com que frequência você ou alguém da sua casa foi dormir com sede?*
None	In the last 4 weeks, how frequently has there been no usable or drinkable water whatsoever in your household?	*Sem água*	*Nas últimas 4 semanas, com que frequência você não tem água (nada) em sua casa?*
Shame	In the last 4 weeks, how frequently have problems with water caused you or anyone in your household to feel ashamed/excluded/stigmatized?	*Vergonha*	*Nas últimas 4 semanas, com que frequência os problemas com água fizeram com que você ou alguém em sua casa se sentisse envergonhado/excluído/estigmatizado?*

Source: prepared by the authors, based on Young et al. ^15^.

Note: the responses to items are: never (0 times), rarely (1-2 times), sometimes (3-10 times), often (11-20 times), always (more than 20 times), don’t know and not applicable/I don’t have this. Never is scored as 0, rarely is scored as 1, sometimes is scored as 2 and often/always are scored as 3.

In 2023, researchers and policy makers from different organizations met at an international conference in Mexico City (Mexico) to discuss the use of water insecurity scales in Latin America and the Caribbean ^17^. This group, which included Brazilian researchers, identified the need for conducting new studies related to the simultaneous use of water insecurity and food insecurity scales to monitor SDGs and the validation process of water insecurity measurement scales in Portuguese/Spanish in Latin America and the Caribbean to help countries monitor and potentially meet the 2030 SDGs. Additionally, the group suggested the need to evaluate the intersection between water insecurity and food insecurity, both captured by experiential scales at the household level ^15^.

Thus, for Brazilian researchers, it became essential to conduct further validation studies to refine the validity and reliability of scales that can be used to assess and monitor water insecurity in Brazil. Hence, it is encouraging that HWISE has now been applied in several studies in the country, being simultaneously applied with EBIA in a nationally representative survey.

The process of introducing and validating experiential food insecurity scales has strongly informed a similar process for water insecurity experiential scales based on HWISE ^12^ ^,^ ^15^ ^,^ ^16^. To date, four studies have used the current Portuguese version of HWISE (Box 1) to assess water insecurity in Brazil ^18^ ^,^ ^19^ ^,^ ^20^ ^,^ ^21^. Tomaz et al. ^18^ conducted exploratory analyses of the Portuguese HWISE version in the semi-arid region of Ceará and observed a correlation between water insecurity and social vulnerability. In the *II National Survey on Food Insecurity in the COVID-19 Scenario* ^19^, 42% of households experienced water insecurity and severe food insecurity. Mata et al. ^20^ observed 46.1% of water insecurity in the Western Amazon Basin, connected to food insecurity and social inequalities. In a survey conducted in the city of Rio de Janeiro ^21^, water insecurity was also correlated with food insecurity and other social inequities.

Notably, regarding the validation process of the Portuguese version of HWISE, population studies carried out so far have not yet conducted the cross-cultural validation of the scale questions. Furthermore, there is a need to conduct more in-depth psychometric analyses of the internal and external validity of the current Portuguese version of the HWISE. Thus, aiming to develop a valid *Brazilian Household Water Insecurity Scale* for national studies, researchers at various Brazilian universities are exploring the validity of the current Portuguese HWISE to assess water insecurity across different regions and biomes in Brazil. The HWISE version under study contains 12 items, each with five response options and a recall period of four weeks (Box 1) covering dimensions of water access in households. A household water insecurity score is computed by adding the scores from each of the 12 items within a possible range from 0 to 36 ^22^. The scores 0-2, 3-11, 12-23, and 24-36 represent none-to-marginal, low, moderate, and severe water insecurity, respectively ^22^.

Given the successful Brazilian experience in developing and using EBIA ^7^, it is expected that validating a *Brazilian Household Water Insecurity Scale* in urban and rural settings, as well as different biomes, will strongly improve the formulation of public policies aimed at ensuring the right to adequate access to water and mitigate the negative effects of climate change on the country’s food security status. Furthermore, it is necessary to advance the debate on the connections between water insecurity and social inequalities, which are also related to food insecurity ^23^.

Hence, following the footsteps of EBIA in monitoring hunger in the country, incorporating a valid and reliable scale to assess water and food insecurity simultaneously in national studies should enable monitoring of hunger dimensions influenced by different levels of water access. As such, the country will continue to lead in Latin America and the Caribbean ^23^ on addressing current public health challenges by monitoring and acting on the social determinants of health, including food insecurity and water insecurity ^8^ ^,^ ^16^. This will also enable better monitoring of the country’s progress towards the SDGs, making corrections and adjustments as needed via policies and programs.
